# Three-Dimensional Pelvic Kinematics During Direct Anterior Approach Total Hip Arthroplasty on an Orthopaedic Table

**DOI:** 10.1016/j.artd.2026.101965

**Published:** 2026-02-25

**Authors:** Kathryn H. Colone, Jacqueline E. Wright, Daniele Marras, Ryan C. Knowles, Casey A. Myers, Joseph B. Assini, Chadd W. Clary

**Affiliations:** a Center for Orthopaedic Biomechanics, University of Denver, Denver, CO, USA; b Mizuho OSI, Union City, CA, USA; c Swedish Medical Center, Englewood, CO, USA

**Keywords:** Total hip arthroplasty, Direct anterior approach, Traction table, Pelvic kinematics

## Abstract

**Background:**

Intraoperative pelvic positioning plays an important role in component placement during total hip arthroplasty (THA), affecting range of motion, stability, and dislocation risk. The objective of this study was to evaluate three-dimensional pelvic kinematics during several key surgical steps of direct anterior approach (DAA) THA facilitated by a Hana table.

**Methods:**

DAA THA was performed on 10 hips from 5 cadaveric specimens. Pelvis and table movement were recorded throughout the procedure using motion capture arrays. Bony anatomy was registered to computed tomography–based segmentations to establish meaningful kinematic data. Pelvic flexion, lateral tilt (LT), and axial rotation (AR) were reported relative to the Hana table.

**Results:**

The pelvis was oriented anteriorly in the initial supine position, with minimal LT (median: 0.1°, range: −5.7° to 5.0°) and AR (median: −1.3°, range: −3.6° to 3.6°). Minimal pelvic flexion was observed across all surgical steps of the procedure, while higher magnitudes of LT and AR were exhibited. Lateral tilt and AR generally occurred toward the operative hip, except for AR away from the operative hip during acetabular reaming.

**Conclusions:**

These measurements suggest it may be necessary for surgeons to consider intraoperative pelvic tilt during cup positioning. This study offers a comprehensive set of pelvic kinematics throughout DAA THA, offering valuable insights for surgical decision-making and enhancing component positioning.

## Introduction

The positioning of the acetabular cup is a critical aspect of total hip arthroplasty (THA), as suboptimal positioning is associated with reduced hip range of motion, instability, poor patient satisfaction, accelerated implant wear, and dislocation risk [[1], [2], [3], [4], [5], [6], [7], [8]]. Intraoperative pelvic positioning affects the orientation of the acetabulum and the final placement of the implanted cup. Several studies have examined the effects of pelvic rotations on the functional orientation of the acetabular cup. For each degree of pelvic flexion (PF) in the sagittal plane, there is a 0.5° to 1° change in anteversion and 0.2° change in inclination of the acetabulum [[9], [10], [11], [12], [13], [14], [15]]. Lateral tilt (LT) of the pelvis in the coronal plane directly affects inclination, while each degree of axial rotation (AR) of the pelvis in the transverse plane results in 0.6° to 1° change in acetabular anteversion and 0.2° change in inclination [9,11]. These relationships have important implications for the target component placement.

Studies have investigated the effects of patient positioning and surgical approach on pelvic orientation to better understand the accuracy of acetabular component orientation. Direct anterior approach (DAA), performed supine, is a common technique for THA that has gained popularity due to its muscle-sparing nature [16]. Although many studies have reported favorable outcomes with DAA [7,[17], [18], [19]], variability in supine pelvic orientation is reportedly high. Studies have reported supine pelvic tilt from preoperative CT scans ranging from −35.0° to 24.5° [[20], [21], [22]]. This variability contributes to an ongoing debate over the optimal patient position during THA.

Orthopaedic traction tables are patient positioning devices designed to enhance the DAA THA procedure by facilitating lower extremity manipulation and assisting with joint exposure [[23], [24], [25]]. However, there is limited data regarding intraoperative pelvic positioning on an orthopaedic table. Roettges et al. quantified pelvic tilt preoperatively as well as intraoperatively on a Hana table (Mizuho OSI, Union City, CA) after reduction of the hip. Pelvic tilt was significantly lower intraoperatively compared to the preoperative radiographs in a “consistent and predictable” manner [26]. The majority of similar studies report intraoperative pelvic positioning at isolated time points, typically after hip reduction. Consequently, the literature lacks comprehensive measurements of three-dimensional pelvic orientation throughout the DAA THA procedure, specifically during steps critical to implant positioning [[26], [27], [28], [29]]. Therefore, the objective of this study was to quantify three-dimensional pelvic kinematics during several key steps of DAA THA facilitated by a Hana table.

## Material and methods

**Figure 1:**
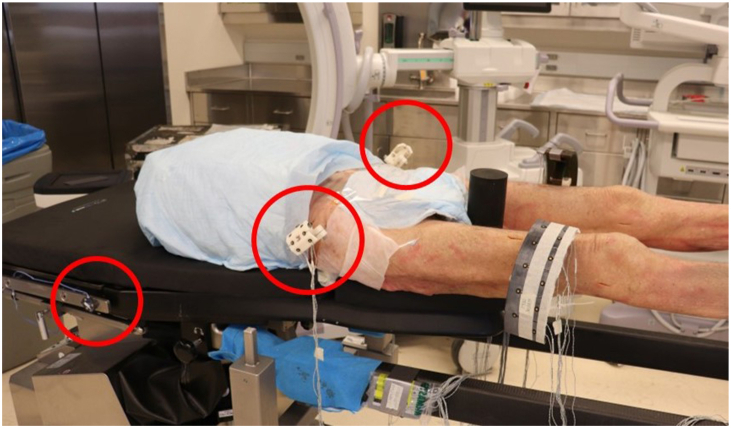
Specimen positioned supine on the Hana table with tracking arrays rigidly attached to the pelvis and Hana table, indicated with red circles.

**Figure 2:**
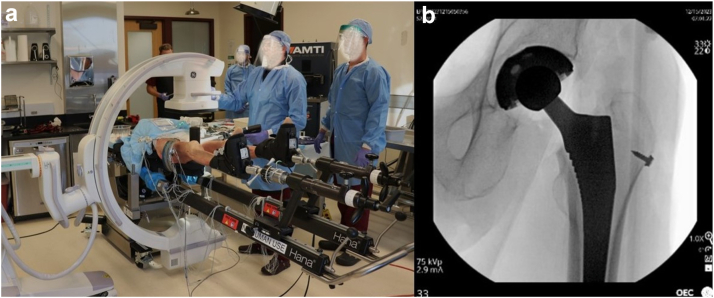
(a) C-arm configuration during THA. (b) Representative C-arm image after final reduction of the implanted hip.

Ten hips from 5 torso-to-toe cadaveric specimens were thawed for 48 hours and tested (4 males, 1 female; 74.6 ± 13.3 years; body mass index (BMI): 18.8 ± 4.1 kg/m^2^). Prior to testing, high-resolution CT scans were performed, and the bony anatomy of the pelvis was segmented. Specimens were positioned supine on the Hana table with the feet secured in the leg spars. Tracking arrays were rigidly attached to the pelvis and Hana table to record kinematics using an optical camera system, which provides submillimeter positional accuracy (Optotrak Certus, Northern Digital Inc, Waterloo, ON) (Fig. 1). DAA THA procedures were performed by a single surgeon (J.B.A.) using the perineal post. All hips were implanted with Corail stems and Pinnacle cups (DePuy Synthes, Warsaw, IN), and a C-arm was used intraoperatively to confirm implant alignment (General Electric, Boston, MA) (Fig. 2).

**Figure 3:**
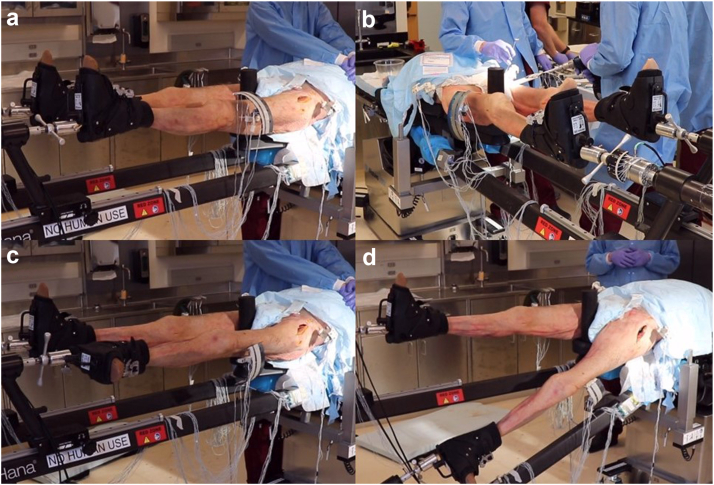
Representative leg spar positions associated with kinematic measurements: (a) neutral position, (b) acetabular reaming with neutral leg spar positioning, (c) external rotation of the operative leg (Movement 1), and (d) hyperextension and adduction of the operative leg (Movement 2).

Pelvis and table kinematics were evaluated during each surgical step associated with DAA THA on a Hana table (Fig. 3), including:

1. The initial neutral positioning of the specimen on the table
2. During and after acetabular reaming
3. After external rotation of the operative leg with partial hyperextension of the hip using the spar to facilitate soft tissue releases (Movement 1)
4. After full hyperextension of the hip and adduction under the contralateral leg to facilitate access to the resected calcar for broaching (Movement 2)
5. After femoral broaching was completed
6. After flexion and abduction of the hip back to the neutral position (Movement 3)
7. During and after reduction of the implanted hip, involving inferior distraction and internal rotation of the leg (Movement 4)

For acetabular reaming, the first reaming sequence was analyzed, corresponding to the initial reamer size used during the procedure. Pelvic kinematics during reaming were quantified at 2 time points: 1) the peak excursion during reaming, marked by the most medial pelvic translation, and 2) the pelvic position after the reamer was removed. Prescribed external rotation of the operative leg during the procedures (Movement 1) ranged from 100° to 120°, based on surgeon discretion.

After surgery, fiducial markers were placed in the pelvis and digitized using the motion capture system. The pelvis was denuded and optically scanned (Space Spider, Artec 3D, Senningerberg, Luxembourg). The optical scanner had a reported accuracy of 0.05 mm. The scanned fiducial markers were aligned to the digitized fiducials from the optical tracking data using singular value decomposition. Residual differences between the scanned and digitized fiducial markers after registration were quantified using the resulting root mean square difference, which averaged 1.2 mm ± 1.2 mm across specimen.

A pelvic anatomic coordinate system was established using the CT-based segmentations (ScanIP, Simpleware Inc., Sunnyvale, CA) [30]. The origin was established at the center of the operative acetabulum. The anterior–posterior (AP) direction was perpendicular to the anterior pelvic plane, a plane connecting the left and right anterior superior iliac spines and the midpoint of the pubic tubercles. The medial–lateral (ML) axis was parallel to the line connecting the left and right anterior superior iliac spine points, directed to the right. The superior–inferior (SI) axis was mutually orthogonal to the AP and ML axes, parallel to the anterior pelvic plane. A separate coordinate system was aligned to the Hana table to define the orientation of the pelvis relative to the table in the initial position.

**Figure 4:**
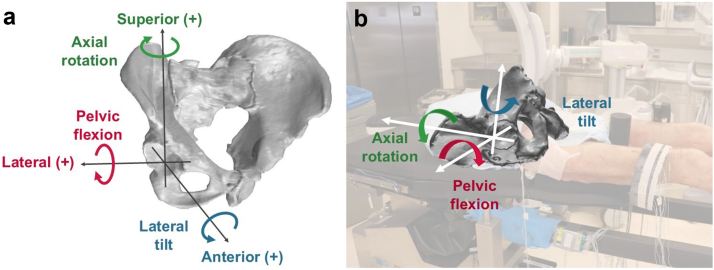
(a) Sign conventions for pelvic kinematics. Anterior pelvic flexion, lateral tilt toward the operative hip, and pelvic rotation toward the operative hip are positive rotations. Lateral, anterior, and superior translation are positive translations. (b) Rotational degrees of freedom illustrated on the Hana table as the motions are observed during surgery.

Pelvic kinematics were calculated for each surgical step relative to the initial position. Pelvic rotations were reported as pelvic flexion occurring in the sagittal plane, lateral tilt in the coronal plane, and axial rotation in the transverse plane (Fig. 4). Pelvic translations were reported along the pelvic ML, AP, and SI axes relative to the initial position.

Left and right hips were treated as independent observations, as each hip underwent a distinct surgical procedure. To assess potential correlation between left and right hips, Spearman rank correlation analyses were performed across all surgical steps and kinematic degrees of freedom. No significant correlations were observed between right and left hips (*P* > .05). Friedman's nonparametric test was used to compare surgical steps for each kinematic degree of freedom. Effect sizes were quantified using Kendall’s coefficient of concordance (W). For significant results, post hoc pairwise comparisons were conducted using rank-based tests with Bonferroni correction to identify differences between surgical steps (α = 0.05).

## Results

**Figure 5:**
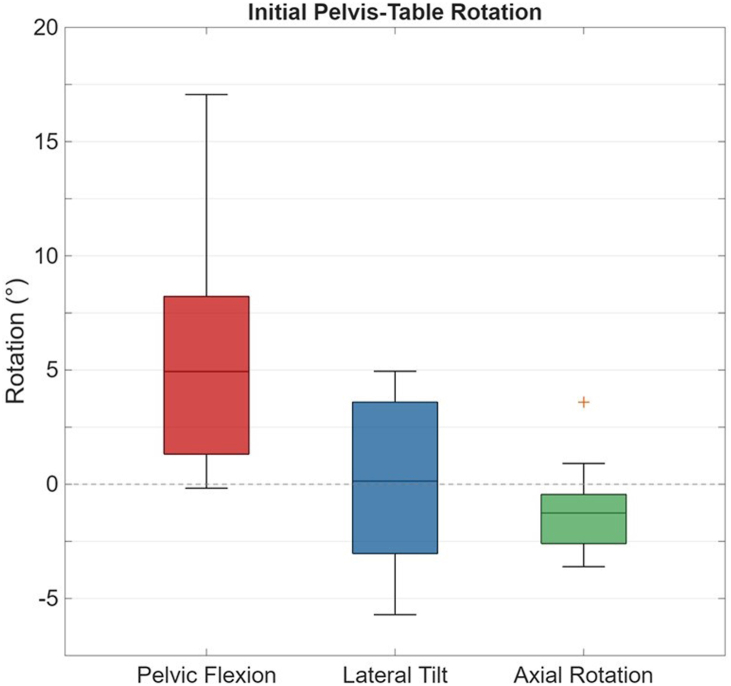
Initial pelvis-table position, defined as the rotation of the pelvis relative to the table at the start of surgery. Dotted horizontal line indicates 0°, representing no relative rotation between the pelvis and the table.

Surgeries were performed on all 10 hips; however, pelvic kinematics were only reported for 9 out of 10 hips due to a faulty tracking array that resulted in corrupted data for one specimen. In the initial supine position, the pelvis was oriented with anterior pelvic flexion relative to the table (median: 4.9°, range: −0.2° to 17.1°), and with negligible lateral tilt (median: 0.1°, range: −5.7° to 5.0°), and axial rotation (median: −1.3°, range: −3.6° to 3.6°) (Fig. 5).

**Figure 6:**
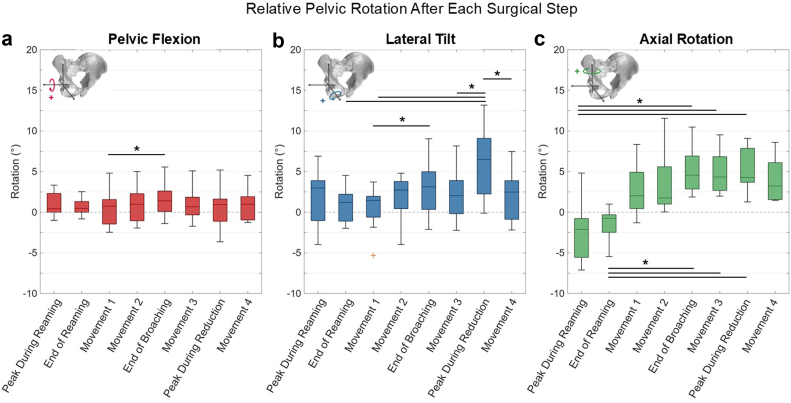
(a) Pelvic flexion, (b) lateral tilt, and (c) axial rotation after each of the surgical steps, relative to the start of surgery. ∗*P* < .05 between surgical steps. Dotted horizontal line indicates 0°, representing no rotation relative to the initial position. Inset figures illustrate the positive rotation for each respective degree of freedom.

**Figure 7:**
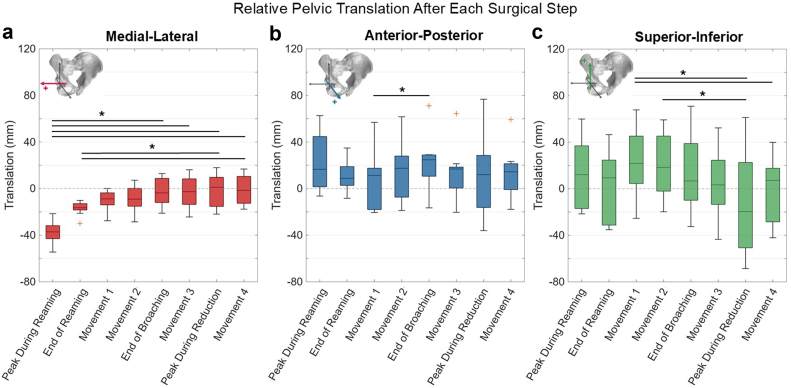
(a) Medial–lateral, (b) anterior–posterior, and (c) superior–inferior pelvic translation after each of the surgical steps, relative to the start of surgery. ∗*P* < .05 between surgical steps. Dotted horizontal line indicates 0 mm, representing no translation relative to the initial position. Inset figures illustrate the positive translation for each respective degree of freedom.

The largest changes in pelvic kinematics were observed in lateral tilt, axial rotation, and medial-lateral translation (*P* < .001, Kendall’s W > 0.5), with smaller changes in pelvic flexion, anterior-posterior translation, and superior-inferior translation between surgical steps (*P* < .05, Kendall’s W > 0.2) (Table 1). During reaming, peak pelvic axial rotation (median: −2.1°, range: −7.1° to 4.8°) was coupled with lateral tilt (median: 3.0°, range: −4.0° to 6.9°), retaining some residual rotations after reaming was complete (Fig. 6, Table 2). Axial rotations measured during reaming were significantly different from several other surgical steps (*P* < .001) (Fig. 6, Table 2). Minimal pelvic flexion (median: 0.4°, range: −1.0° to 3.3°) was observed during reaming. Median peak ML, AP, and SI translations were −37.2 mm (range: −54.5 mm to −21.5 mm), 16.6 mm (range: −6.4 mm to 62.7 mm), and 12.1 mm (range: −21.6 mm to 59.9 mm), respectively, with minor residual translations after reaming (Fig. 7, Table 3). The medial translations measured during reaming were the largest throughout the procedure, differing significantly from the surgical steps following femoral broaching (*P* < .05). The maximum SI translations during the procedure occurred after Movement 1 (median: 21.7 mm; range: −25.4 mm to 67.8 mm).

**Table 1:** Friedman test statistics and Kendall's W for pelvic kinematic degrees of freedom.

Degree of freedom	Friedman's χ2	*P* value	Kendall's W
Pelvic flexion	15.0	.036	0.238
Lateral tilt	13.7	<.001	0.502
Axial rotation	45.4	<.001	0.721
Medial–lateral translation	45.8	<.001	0.727
Anterior–posterior translation	15.8	.027	0.25
Superior–inferior translation	29.1	<.001	0.463

**Table 2:** Pelvic rotations after each surgical step, relative to the initial pelvic position.

Surgical step	Pelvic rotation (degrees)		
	Pelvic flexion	Lateral tilt	Axial rotation
Peak during reaming	0.4 (−1.0 to 3.3)^a^	3.0 (−4.0 to 6.9)	−2.1 (−7.1 to 4.8)
End of reaming	0.5 (−0.8 to 2.5)	1.2 (−2.0 to 4.5)	−0.7 (−5.5 to 1.0)
Movement 1	0.7 (−2.5 to 4.8)	1.5 (−5.3 to 3.7)	2.0 (−1.3 to 8.3)
Movement 2	1.0 (−1.9 to 5.0)	2.7 (−4.0 to 4.8)	1.8 (0 to 11.6)
End of broaching	1.4 (−1.4 to 5.6)	3.1 (−2.1 to 9.0)	4.6 (1.9 to 10.5)
Movement 3	0.7 (−1.7 to 5.1)	2.0 (−2.2 to 8.2)	4.3 (2.0 to 9.5)
Peak during reduction	0.9 (−3.6 to 5.2)	6.5 (−0.1 to 13.2)	4.3 (1.3 to 9.1)
Movement 4	1.0 (−1.3 to 4.5)	2.5 (−2.2 to 7.5)	3.2 (1.5 to 8.6)

a Values reported as median; range.

**Table 3:** Pelvic translations after each surgical step, relative to the initial pelvic position.

Surgical step	Pelvic translation (mm)		
	Medial–lateral	Anterior–posterior	Superior–inferior
Peak during reaming	−37.2 (−54.5 to −21.5)^a^	16.6 (−6.4 to 62.7)	12.1 (−21.6 to 59.9)
End of reaming	−16.5 (−29.9 to 10.2)	8.8 (−8.3 to 34.8)	9.3 (−35.2 to 46.5)
Movement 1	−8.8 (27.6 to 0.1)	11.3 (−20.5 to 56.9)	21.7 (−25.4 to 67.8)
Movement 1	−9.1 (−28.5 to 7.2)	17.4 (−18.8 to 61.7)	18.3 (−19.8 to 59.2)
End of broaching	−3.7 (−21.1 to 13.0)	24.7 (−16.5 to 71.1)	6.7 (−32.5 to 70.9)
Movement 3	−2.7 (−24.4 to 16.2)	16.9 (−20.4 to 64.4)	3.3 (−43.5 to 52.3)
Peak during reduction	1.0 (−21.9 to 18.0)	11.9 (36.1 to 76.7)	−19.6 (−68.6 to 61.3)
Movement 4	−1.5 (−17.7 to 16.8)	14.4 (−17.8 to 59.3)	7.2 (−42.1 to 39.8)

a Values reported as median (range).

Median lateral tilt and axial rotation increased with Movement 2 and femoral broaching relative to the pelvic position at the start of surgery (Fig. 6, Table 2). Lateral tilt and axial rotation decreased during Movement 3 as the hip was returned to the neutral position, although this change was not statistically significant. The maximum observed lateral tilt (median: 6.5°; range: −0.1° to 13.2°) occurred during reduction of the femur, accompanied by similar magnitude of axial rotation (median: 4.3°; range: 1.3° to 9.1°) (Fig. 6, Table 2). The peak lateral tilt observed during reduction was significantly different from lateral tilt during several other surgical steps (*P* < .05) (Fig. 6).

## Discussion

There is limited data on intraoperative pelvic positioning during the surgical motions of DAA THA on a traction table. This study quantified changes in pelvic kinematics at each step of the procedure. Our results showed that throughout the DAA procedure, the pelvis generally tilted anteriorly and rotated toward the operative hip in the axial and coronal planes. Kendall’s W effect sizes indicated that several pelvic degrees of freedom demonstrated moderate to strong agreement throughout the procedure, suggesting that changes in pelvic motion followed consistent, surgical step-dependent patterns (Table 1).

Pelvic flexion remained minimal across the procedure, with less than 2° average pelvic flexion relative to the initial position. The lateral tilt toward the operative hip increased after Movement 1 through femoral broaching and returned toward the neutral position after reduction of the implanted hip. Axial rotation also increased toward the operative hip after Movement 1 through femoral broaching and remained elevated through the end of the procedure (Fig. 6). The magnitudes of pelvic flexion and axial rotation in this study were consistent with those reported in previous studies on standard operating tables [28,31]. After reaming, the maximum observed pelvic rotation was 5.5° axial rotation toward the nonoperative hip, which may cause a 5.5° change in acetabular anteversion and 1.1° change in inclination. These changes in acetabular anteversion and inclination, resulting from movement of the pelvis, may be clinically relevant depending on when cup alignment is assessed [[32], [33], [34]].

Reports of preoperative and intraoperative pelvic flexion vary widely in the literature. In studies by LeBrun et al. and Yun et al., preoperative supine pelvic flexion was reported as 4.0° ± 7.0° and −2.2° ± 7.7°, respectively [21,22]. Mouri et al. reported neutral pelvic flexion (0° ± 0.5°) intraoperatively on standard operating tables, while Okamoto et al. reported initial posterior pelvic flexion (−7.4°) [28,31]. In our study, pelvic flexion in the initial position on a Hana table was 6.0° ± 5.7°, indicating a more anteriorly oriented pelvis than previous studies. In the final position after reduction, pelvic kinematics were similar to those reported by previous studies. Roettges et al. reported 3.0° ± 6.2° pelvic flexion on a Hana table, whereas our study measured 0.8° ± 1.9° pelvic flexion indicating similar magnitudes with less variability. Differences in the initial pelvic flexion in our study could be attributed to using cadaveric tissue or natural variability between subjects and a small sample size. Our data indicate minimal changes in pelvic flexion throughout the procedure, which may allow clinicians to compensate for the acetabular cup orientation if the initial pelvic flexion is known. Notably, hyperextension of the hip during Movements 1 and 2 did not significantly affect pelvic flexion. In contrast, pelvic flexion in the lateral decubitus position for posterior approach THA reportedly ranges −25° to 20° [35], with pelvic rotation between initial set-up and cup placement of 9.0° ± 6.0° [36]. The increased variability in pelvic positioning during posterior approach THA makes accurate acetabular cup orientation more difficult.

Variability in pelvic axial rotation during THA has recently gained attention for its role in accurate acetabular cup placement. Aichmair et al. quantified axial rotation of the pelvis supine on a traction table, reporting 3.5° ± 2.1° (range: −1.6° to 8.5°) rotation toward the operative hip [27]. Using surgical navigation, Mouri et al. reported 0.4° ± 2.0° axial rotation at skin incision, which increased to 4.6° ± 3.9° of rotation toward the operative hip at cup implantation on a standard operating table. In our study, we observed axial rotation of −1.1° ± 2.2° (range: −3.6° to 3.6°) in the initial position, which rotated away from the operative hip during reaming (−2.5° ± 3.7°) and toward the operative hip during leg spar manipulation and femoral broaching (5.2° ± 3.0°). Depending on when the surgeon performs cup impaction, these variations could cause significant changes in the functional alignment of the hip. Advancements in surgical robotics, navigation technologies, and intraoperative imaging enabled by radiolucent table systems may negate the clinical impacts of this variability.

This study was conducted by a single surgeon using DAA and a single implant type on a Hana table, limiting generalizability to other traction tables, instrument systems, or surgical techniques. Additionally, this study includes a small sample size of low BMI cadaveric specimens, so pelvic movements may differ in living and high BMI patients [31,37].

## Conclusions

This study provided a comprehensive quantitative analysis of three-dimensional pelvic motions during DAA THA. These results indicated axial rotations and lateral tilt toward the operative hip during leg spar movements, with the exception of acetabular reaming. These findings offer valuable insights for surgeons placing components during surgical steps with higher pelvic rotations. Considering these intraoperative pelvic rotations may enhance the accuracy of component positioning in THA.

## Funding

This study was funded in part by Mizuho OSI, which contributed to the study design. The sponsor did not influence the analysis, interpretation, or reporting of the results.

## CRediT authorship contribution statement

**Kathryn H. Colone:** Writing – review & editing, Writing – original draft, Visualization, Validation, Supervision, Software, Resources, Project administration, Methodology, Investigation, Formal analysis, Data curation. **Jacqueline E. Wright:** Writing – review & editing, Writing – original draft, Visualization, Methodology, Funding acquisition, Data curation, Conceptualization. **Daniele Marras:** Methodology, Investigation, Data curation. **Ryan C. Knowles:** Investigation, Data curation. **Casey A. Myers:** Writing – review & editing, Supervision, Project administration, Methodology, Funding acquisition, Data curation, Conceptualization. **Joseph B. Assini:** Writing – review & editing, Methodology, Investigation, Data curation. **Chadd W. Clary:** Writing – review & editing, Writing – original draft, Supervision, Project administration, Methodology, Investigation, Funding acquisition, Formal analysis, Data curation, Conceptualization.

## Conflicts of interest

C.W. Clary received research support from Depuy Synthes as a Principal Investigator. C.A. Myers received royalties from Depuy Synthes; owns stock or stock options in Ortho Haus, LLC; and received research support from Mizuho, OSI; Depuy Synthes as a Principal Investigator. J.B. Assini received royalties from Microport Orthopedics; received speakers' bureau/paid presentations for Microport Orthopedics; is a paid consultant for Microport Orthopedics; and received research support from Microport Orthopedics, Sarah Cannon Research as a Principal Investigator; all other authors declare no potential conflicts of interest.

For full disclosure statements refer to https://doi.org/10.1016/j.artd.2026.101965.

## Appendix A Supplementary Data

Conflict of Interest Statement for Clary

Conflict of Interest Statement for Myers

Conflict of Interest Statement for Marras

Conflict of Interest Statement for Assini

Conflict of Interest Statement for Wright

Conflict of Interest Statement for Colone

Conflict of Interest Statement for Knowles
