# Supplementary material for: Three-Dimensional Pelvic Kinematics During Direct Anterior Approach Total Hip Arthroplasty on an Orthopaedic Table
Source: Arthroplast Today. 2026 Feb 25;38:101965. doi: 10.1016/j.artd.2026.101965 (PMC12954283; doi:10.1016/j.artd.2026.101965)
Supplement: Conflict of Interest Statement for Assini [file mmc4.pdf]

INDIVIDUAL CONFLICT OF INTEREST STATEMENT

American Association of Hip and Knee Surgeons

(Adopted from the American Academy of Orthopaedic Surgeons disclosure statement)

The following form **must be filled out completely and submitted by each author (example, 6 authors, 6 forms). All items require a response. If there is no relevant disclosure for a given item, enter "*None.*"**

Manuscript Title Three-Dimensional Pelvis Kinematics During Direct Anterior Approach Total Hip Arthroplasty on an Orthopaedic Table

1. Royalties from a company or supplier (The following conflicts were disclosed)
   - Microport Orthopedics

2. Speakers bureau/paid presentations for a company or supplier (The following conflicts were disclosed)
   -Microport Orthopedics

3A. Paid employee for a company or supplier (The following conflicts were disclosed)
   -None

3B. Paid consultant for a company or supplier (The following conflicts were disclosed)
   -Microport Orthopedics

3C. Unpaid consultants for a company or supplier (The following conflicts were disclosed)
   -None

4. Stock or stock options in a company or supplier (The following conflicts were disclosed)
   -None

5. Research support from a company or supplier as a Principal Investigator (The following conflicts were disclosed)
   -Microport Orthopedics, Sarah Cannon Research

6. Other financial or material support from a company or supplier (The following conflicts were disclosed)
   -None

7. Royalties, financial or material support from publishers (The following conflicts were disclosed)
   -None

8. Medical/Orthopaedic publications editorial/governing board (The following conflicts were disclosed)
   -None

9. Board member/committee appointments for a society (The following conflicts were disclosed)
   -None

Each author must sign AND print or type his/her name, date and submit a separate form

In addition, one BLINDED Conflict of Interest form (no author names used) should be submitted per manuscript with all author disclosures.

J. ASSINI [signature] 4/1/25

Author Name (Print or Type) | Author Signature | Date
