# Supplementary material for: Three-Dimensional Pelvic Kinematics During Direct Anterior Approach Total Hip Arthroplasty on an Orthopaedic Table
Source: Arthroplast Today. 2026 Feb 25;38:101965. doi: 10.1016/j.artd.2026.101965 (PMC12954283; doi:10.1016/j.artd.2026.101965)
Supplement: Conflict of Interest Statement for Wright [file mmc5.pdf]

INDIVIDUAL CONFLICT OF INTEREST STATEMENT

American Association of Hip and Knee Surgeons

(Adopted from the American Academy of Orthopaedic Surgeons disclosure statement)

The following form **must be filled out completely and submitted by each author (example, 6 authors, 6 forms).**
All items require a response. If there is no relevant disclosure for a given item, enter "*None.*"

Manuscript Title Three-Dimensional Pelvis Kinematics During Direct Anterior Approach Total Hip Arthroplasty on an Orthopaedic Table

1. Royalties from a company or supplier (The following conflicts were disclosed)

   none

2. Speakers bureau/paid presentations for a company or supplier (The following conflicts were disclosed)

   none

3A. Paid employee for a company or supplier (The following conflicts were disclosed)

   none

3B. Paid consultant for a company or supplier (The following conflicts were disclosed)

   none

3C. Unpaid consultants for a company or supplier (The following conflicts were disclosed)

   none

4. Stock or stock options in a company or supplier (The following conflicts were disclosed)

   none

5. Research support from a company or supplier as a Principal Investigator (The following conflicts were disclosed)

   none

6. Other financial or material support from a company or supplier (The following conflicts were disclosed)

   none

7. Royalties, financial or material support from publishers (The following conflicts were disclosed)

   none

8. Medical/Orthopaedic publications editorial/governing board (The following conflicts were disclosed)

   none

9. Board member/committee appointments for a society (The following conflicts were disclosed)

   none

Each author must sign AND print or type his/her name, date and submit a separate form

In addition, one BLINDED Conflict of Interest form (no author names used) should be submitted per manuscript with all author disclosures.

Jacqueline Wright	[signature]	4/29/25
Author Name (Print or Type)	Author Signature	Date
