# Supplementary material for: Three-Dimensional Pelvic Kinematics During Direct Anterior Approach Total Hip Arthroplasty on an Orthopaedic Table
Source: Arthroplast Today. 2026 Feb 25;38:101965. doi: 10.1016/j.artd.2026.101965 (PMC12954283; doi:10.1016/j.artd.2026.101965)
Supplement: Conflict of Interest Statement for Colone [file mmc6.pdf]

INDIVIDUAL CONFLICT OF INTEREST STATEMENT

American Association of Hip and Knee Surgeons

(Adopted from the American Academy of Orthopaedic Surgeons disclosure statement)

The following form **must be filled out completely and submitted by each author (example, 6 authors, 6 forms). All items require a response. If there is no relevant disclosure for a given item, enter "*None*."**

Manuscript Title Three-Dimensional Pelvis Kinematics During Direct Anterior Approach Total Hip Arthroplasty on an Orthopaedic Table

1. Royalties from a company or supplier (The following conflicts were disclosed)

none

2. Speakers bureau/paid presentations for a company or supplier (The following conflicts were disclosed)

none

3A. Paid employee for a company or supplier (The following conflicts were disclosed)

none

3B. Paid consultant for a company or supplier (The following conflicts were disclosed)

none

3C. Unpaid consultants for a company or supplier (The following conflicts were disclosed)

none

4. Stock or stock options in a company or supplier (The following conflicts were disclosed)

none

5. Research support from a company or supplier as a Principal Investigator (The following conflicts were disclosed)

none

6. Other financial or material support from a company or supplier (The following conflicts were disclosed)

none

7. Royalties, financial or material support from publishers (The following conflicts were disclosed)

none

8. Medical/Orthopaedic publications editorial/governing board (The following conflicts were disclosed)

none

9. Board member/committee appointments for a society (The following conflicts were disclosed)

none

Each author must sign AND print or type his/her name, date and submit a separate form

In addition, one BLINDED Conflict of Interest form (no author names used) should be submitted per manuscript with all author disclosures.

Kathryn Colone	Kathryn Colone	04/29/2025
Author Name (Print or Type)	Author Signature	Date
